# Identifying heart failure dynamics using multi-point electrocardiograms and deep learning

**DOI:** 10.1093/ehjdh/ztaf016

**Published:** 2025-03-10

**Authors:** Yu Nishihara, Makoto Nishimori, Satoki Shibata, Masakazu Shinohara, Ken-Ichi Hirata, Hidekazu Tanaka

**Affiliations:** Division of Cardiovascular Medicine, Department of Internal Medicine, Kobe University Hospital, 7-5-2, Kusunoki-cho, Chuo-ku, Kobe 650-0017, Japan; Division of Molecular Epidemiology, Kobe University Graduate School of Medicine, 7-5-2, Kusunoki-cho, Chuo-ku, Kobe 650-0017, Japan; Kobe University School of Medicine, Kobe University, 7-5-2, Kusunoki-cho, Chuo-ku, Kobe 650-0017, Japan; Division of Molecular Epidemiology, Kobe University Graduate School of Medicine, 7-5-2, Kusunoki-cho, Chuo-ku, Kobe 650-0017, Japan; Division of Cardiovascular Medicine, Department of Internal Medicine, Kobe University Hospital, 7-5-2, Kusunoki-cho, Chuo-ku, Kobe 650-0017, Japan; Division of Cardiovascular Medicine, Department of Internal Medicine, Kobe University Hospital, 7-5-2, Kusunoki-cho, Chuo-ku, Kobe 650-0017, Japan

**Keywords:** Heart failure, Deep learning, Electrocardiogram, Brain natriuretic peptide

## Abstract

**Aims:**

Heart failure (HF) hospitalizations are associated with poor survival outcomes, emphasizing the need for early intervention. Deep learning algorithms have shown promise in HF detection through electrocardiogram (ECG). However, their utility in ongoing HF monitoring remains uncertain. This study developed a deep learning model using 12-lead ECGs collected at 2 different time points to evaluate HF status changes, aiming to enhance early intervention and continuous monitoring in various healthcare settings.

**Methods and results:**

We analysed 30 171 ECGs from 6531 adult patients at Kobe University Hospital. The participants were randomly assigned to training, validation, and test datasets. A Transformer-based model was developed to classify HF status into deteriorated, improved, and no-change classes based on ECG waveform signals at two different time points. Performance metrics, such as the area under the receiver operating characteristic curve (AUROC) and accuracy, were calculated, and attention mapping via gradient-weighted class activation mapping was utilized to interpret the model’s decision-making ability. The patients had an average age of 64.6 years (±15.4 years) and brain natriuretic peptide of 66.3 pg/mL (24.6–175.1 pg/mL). For HF status classification, the model achieved an AUROC of 0.889 [95% confidence interval (CI): 0.879–0.898] and an accuracy of 0.871 (95% CI: 0.864–0.878).

**Conclusion:**

Transformer-based deep learning model demonstrated high accuracy in detecting HF status changes, highlighting its potential as a non-invasive, efficient tool for HF monitoring. The reliance of the model on ECGs reduces the need for invasive, costly diagnostics, aligning with clinical needs for accessible HF management.

**IRB Information:**

Kobe University Hospital Clinical & Translational Research Center (reference number: B220208)

## Graphical Abstract

**Graphical Abstract:**
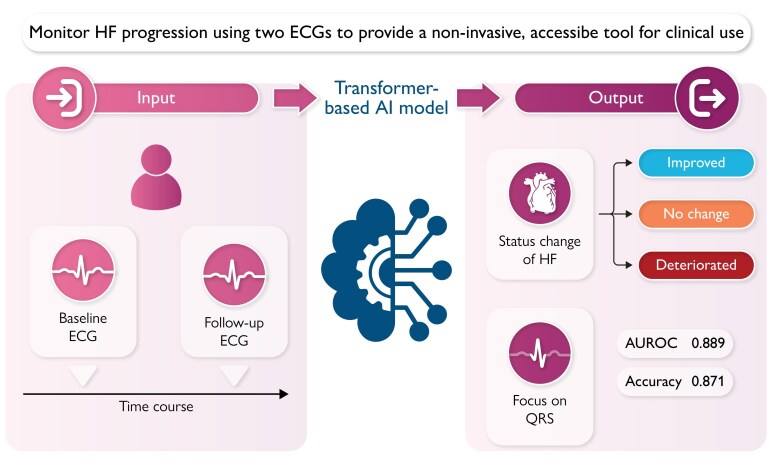
The deep learning model uses paired electrocardiograms (ECGs) to classify heart failure (HF) status (deteriorated, improved, and no-change). This non-invasive tool demonstrates high accuracy and potential for enhanced HF monitoring across healthcare settings.

## Introduction

Heart failure (HF) is a serious health condition associated with a high mortality risk that affects ∼840 out of every 100 000 adults annually, with a 1 year case fatality rate of 24%.^1^ Recent deep learning models have shown promise in non-invasively and rapidly analysing 12-lead electrocardiograms (ECGs) to predict asymptomatic left ventricular ejection fraction decline [area under the receiver operating characteristic curve (AUROC) = 0.93],^2^ left ventricular dysfunction (AUROC = 0.83),^3^ and right ventricular dysfunction (AUROC = 0.84).^4^ However, these models are predicated on an assessment of a single 12-lead ECG, thus limiting their utility in continuous HF monitoring.

The gold standard for monitoring HF includes biomarker evaluations such as brain natriuretic peptide (BNP) tests and echocardiography. These methodologies, however, come with limitations: BNP testing involves blood sampling, making it invasive, and echocardiography’s efficacy heavily relies on the sonographer’s skill. Additionally, access to these diagnostic assessments is not universally available across all healthcare facilities. In clinical practice, BNP testing is often performed in patients suspected of HF, even when values fall below the diagnostic threshold of 100 pg/mL. Such cases are commonly encountered, as BNP testing is frequently used to rule out HF in symptomatic patients.

Given the high recurrence rate and worsening prognosis with repeated HF hospitalizations, timely intervention in deteriorating HF is important.^5–7^ This emphasizes the need for an accessible, cost-effective, and rapid tool to accurately detect changes in HF status.

To address this challenge, we developed a deep learning model that utilizes waveforms from 12-lead ECGs administered at 2 separate time points. This model aims to assess whether HF has worsened or improved, offering a potential breakthrough in HF management. This approach would enable any healthcare facility with access to 12-lead ECGs to conveniently and efficiently monitor changes in HF status, thus advancing the capability for early intervention and continuous patient monitoring across various settings.

## Methods

### Study design, data sources, and patient population

**Figure 1:**
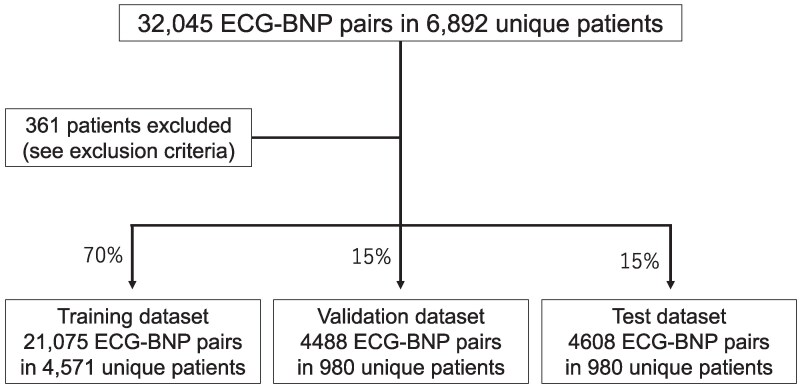
Patient selection and data distribution. A total of 32 045 electrocardiogram–brain natriuretic peptide pairs from 6892 patients were initially identified. Patients were included if they had at least 2 paired 12-lead electrocardiogram and brain natriuretic peptide tests performed on the same day on separate occasions. Exclusion criteria included sacubitril/valsartan use and history of sepsis, hyperthyroidism, liver cirrhosis, end-stage renal disease, pulmonary embolism, or morbid obesity (body mass index >35). After exclusions, the remaining data were randomly allocated to training (70%), validation (15%), and test (15%) datasets.

We enrolled 6892 adult patients aged 18 years and above who underwent ECGs and BNP blood tests at the Department of Cardiology, Kobe University Hospital, from January 2012 to December 2022. For inclusion, patients underwent these tests on the same day for each patient, with at least two such paired tests conducted on separate occasions. Exclusion criteria included conditions known to significantly influence BNP levels independently of HF status: sepsis, hyperthyroidism, liver cirrhosis, end-stage renal disease, pulmonary embolism, use of sacubitril/valsartan, or morbid obesity (body mass index >35) and were applied throughout the study period to ensure consistent eligibility. A total of 361 patients were excluded, leaving 6531 patients with 30 171 paired ECG–BNP records for analysis (*Figure 1*). Our clinical study was conducted in accordance with the relevant guidelines and was approved by the Ethics Committee of the Graduate School of Medicine at Kobe University (approval no. B220208) on 22 February 2023, consistent with the principles of the Declaration of Helsinki.

### Assessment for progression of heart failure

In this study, we defined changes in HF states based on the difference between baseline and follow-up BNP values as described below:

1. Improved class: cases meeting both of the following criteria: relative BNP change ≤−40% (i.e. a decrease of 40% or more) and baseline BNP value ≥100 pg/mL.
2. Deteriorated class: cases meeting both of the following criteria: relative BNP change ≥+40% (i.e. an increase of 40% or more) and follow-up BNP value ≥100 pg/mL.
3. No-change class: all cases not meeting the criteria for either the improved or deteriorated class.

Relative BNP change was calculated as: (follow-up BNP−baseline BNP)/baseline BNP × 100%.

The 40% and 100 pg/mL thresholds, supported by recent literature, reflect clinically relevant criteria for detecting HF status changes.^8^

#### Measurement for brain natriuretic peptide level

In Kobe University Hospital, BNP levels were measured in picograms per millilitre (pg/mL). For integration into our model, BNP values underwent a logarithmic transformation.

#### Electrocardiogramdata and predictor variables

The ECG waveform signals were sourced from a 12-lead electrocardiogram and digitized into CSV format. The ECGs had a sampling rate of 500 Hz and a resolution of 1.25 µV, with a 150 Hz low-pass filter.

### Data pre-processing

A total of 6531 participants in this study were randomly assigned to 3 datasets: training (4571 patients, 70%), validation (980 patients, 15%), and test (980 patients, 15%) datasets (*Figure 1*). This approach follows the Transparent Reporting of a Multivariable Model for Individual Prognosis or Diagnosis + artificial intelligence (AI) statement guidelines, ensuring a rigorous and transparent evaluation of the model’s performance.^9^  *Table 1* presents a summary of the baseline characteristics of the patients in each dataset. Random entries from two distinct time points were selected for each learning iteration. When two pairs of ECG waveforms and BNP levels were collected from a single patient, the median duration between the recording dates of the pre-ECG–BNP pair and the post-ECG–BNP pair was determined to be 172 days. A total of 30 171 ECG–BNP pairs were obtained from 6531 patients. For model development and evaluation, we generated all possible combinations of baseline and follow-up ECG–BNP pairs, resulting in a total of 116 858 combinations. The number of combinations per patient was calculated as *m*(*m*−1)/2, where *m* is the number of ECG–BNP measurements for that patient. These combinations were distributed across datasets as follows: training: 83 748 (77.5%), validation: 16 418 (15.0%), and test: 16 692 (20.9%). The class distribution in each dataset was as follows: training—deteriorated 16 037 (19.15%), improved 11 834 (14.13%), and no-change 55 877 (66.72%); validation—deteriorated 3726 (22.69%), improved 2361 (14.38%), and no-change 10 331 (62.92%); and test—deteriorated 3268 (19.58%), improved 2708 (16.22%), and no-change 10 716 (64.20%).

**Table 1:** Patient characteristics

	Training dataset	Validation dataset	Test dataset	*P*-value
Patients, *n*	4571	980	980	
ECG–BNP pairs, *n*	21 075	4488	4608	
Age, years	64.8 ± 15.3	64.3 ± 15.8	64.4 ± 15.7	0.570
Age groups, *n* (%)				
18–60	1373 (30.04%)	296 (30.2%)	296 (30.2%)	0.991
61–70	1317 (28.81%)	286 (29.18%)	294 (30.0%)	0.755
71–80	1354 (29.62%)	286 (29.18%)	280 (28.57%)	0.797
81+	527 (11.53%)	112 (11.43%)	110 (11.22%)	0.963
Gender, *n* (%)				0.272
Male	2880 (63.01%)	602 (61.43%)	593 (60.51%)	
Female	1691 (36.99%)	378 (38.57%)	387 (39.49%)	
Height, cm	161.2 ± 10.2	160.8 ± 10.3	160.9 ± 10.2	0.554
Body weight, kg	61.7 ± 13.0	61.5 ± 13.1	60.9 ± 13.6	0.225
Body mass index, kg/m^2^	23.6 ± 3.6	23.6 ± 3.7	23.3 ± 3.8	0.115
Comorbidity, *n* (%)				
Hypertension	3113 (68.1%)	662 (67.55%)	681 (69.49%)	0.668
Diabetes	1315 (28.77%)	258 (26.33%)	285 (29.08%)	0.277
Cardiomyopathy	489 (10.7%)	105 (10.71%)	103 (10.51%)	0.980
Valve disease	898 (19.65%)	207 (21.12%)	180 (18.37%)	0.297
Ischaemic disease	1600 (35.0%)	343 (35.0%)	352 (35.92%)	0.880
Atrial fibrillation	681 (14.9%)	133 (13.57%)	133 (13.57%)	0.368
Lipidaemia	1657 (36.25%)	357 (36.43%)	374 (38.16%)	0.556
BNP, pg/mL	66.4 (25.2–171.4)	63.1 (21.8–180.6)	70.4 (25.9–183.0)	0.944
Haemoglobin, g/dL	13.6 ± 2.0	13.7 ± 1.9	13.5 ± 2.0	0.191
Serum creatinine, mg/dL	1.0 ± 0.9	1.0 ± 1.0	1.0 ± 0.9	0.921
eGFR, mL/min/1.73 m^2^	63.9 ± 21.3	65.1 ± 20.7	63.8 ± 21.9	0.278
BUN, mg/dL	18.1 ± 9.1	17.9 ± 9.0	18.1 ± 8.2	0.887
Serum sodium, mmol/L	140.0 ± 2.6	139.9 ± 2.5	140.0 ± 2.5	0.634
Serum potassium, mmol/L	4.3 ± 0.4	4.2 ± 0.4	4.2 ± 0.4	0.555
HbA1c, %	6.1 ± 0.9	6.1 ± 1.0	6..0 ± 0.9	0.309
LVEF, %	59.2 ± 12.2	59.0 ± 12.0	59.3 ± 12.4	0.882

Data are presented as mean ± standard deviation for normally distributed data and median and inter-quartile range for non-normally distributed data or *n* (%).

ECG, electrocardiogram; BNP, brain natriuretic peptide; Cre, creatinine; eGFR, estimated glomerular filtration rate; BUN, blood urea nitrogen; HbA1c, haemoglobin A1C; LVEF, left ventricular ejection fraction.

In each step of the deep learning model training, a 2 s segment was randomly selected from the 15 s recording period of the 12-lead ECG data, formatted in the CSV. Given a sampling rate of 500 Hz, for every sample in each step, the model randomly learned from a matrix of size (12, 1000), corresponding to the 12 leads of the ECG over the randomly selected 2 s interval. Note that in models using both baseline and follow-up 12-lead ECGs, the ECGs were concatenated, resulting in a total of 24 leads. Consequently, the matrix size for each sample was (24, 1000). As a form of data augmentation, random noise (uniform distribution) was introduced up to a limit of 0.1 mV to all leads after calibrating the ECG data.

### Model development

#### Model for estimating brain natriuretic peptide level

**Figure 2:**
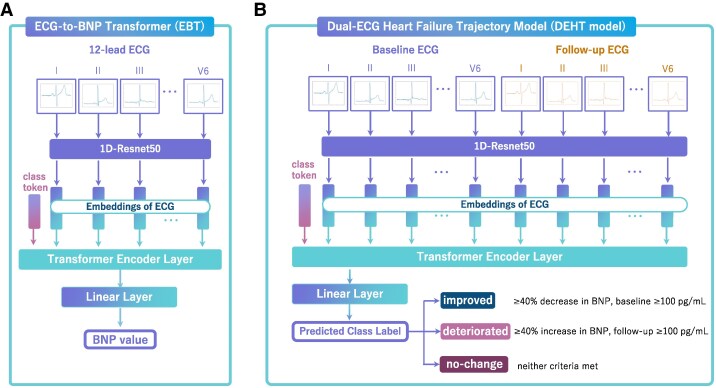
Deep learning models for electrocardiogram analysis and heart failure progression. (*A*) Electrocardiogram-to-brain natriuretic peptide transformer: estimates brain natriuretic peptide levels from a single 12-lead electrocardiogram. The model processes electrocardiogram signals through a convolutional neural network and transformer architecture to predict brain natriuretic peptide values. (*B*) Dual-electrocardiogram heart failure trajectory model: classifies heart failure progression using paired baseline and follow-up electrocardiograms. It analyses changes between two time points to directly categorize patients into either improved (≥40% brain natriuretic peptide decrease, baseline ≥ 100 pg/mL), deteriorated (≥40% brain natriuretic peptide increase, follow-up ≥ 100 pg/mL), or no-change groups.

We developed a model to regress BNP by combining a convolutional neural network (CNN) model based on the ResNet50 architecture with a Transformer model.^10–12^ The CNN utilized the 1D Resnet50 model (1D Resnet50), applying a consistent set of weights across all 12 leads of the ECG to extract feature vectors.^13,14^ This implies that the ECG data from each lead were embedded into the same 256-dimensional feature space. Each of these feature vectors, along with a class token, was then fed into the encoder of the Transformer model, outputting a feature vector derived from the class token. This vector was subsequently input into a linear layer, which predicted the final output, the BNP levels. This architecture, called ‘ECG-to-BNP Transformer (EBT)’, is depicted in *Figure 2A*.

#### Models for detecting progression of heart failure

The EBT model was originally designed to process a single ECG as input and generate estimated values for BNP. Subsequently, the model was adapted to process two timepoint ECGs as input, outputting classifications into three HF classes: improved, no-change, or deteriorated. This alteration means an important transition in the model’s focus, centring on the identification of alterations in states of HF.

For the Transformer model, we adjusted the parameters as follows: a batch size of 64, 8 multi-head attention units, a learning rate varying from 1e−3 to 1e−7 (with a log uniform distribution), a 256-dimensional feature vector, and utilized the Adam optimizer for optimization.

In this study, we developed a dual-ECG heart failure trajectory (DEHT) model, which was trained not on ECG–BNP data but on the pairs of two-point ECGs and the three categories. The DEHT model did not output the raw ΔBNP values; instead, it classified HF status changes (exacerbation or alleviation) based on pre-defined BNP thresholds (≥40% relative change and ≥100 pg/mL) (*Figure 2B*). In this approach, the DEHT model concurrently processed both baseline and follow-up ECGs, employing a singular Transformer model for categorization into one of the three pre-defined classes. This design ensured that the DEHT model operates independently of BNP measurements, relying solely on waveforms of paired ECGs to classify HF status changes.

#### Visualizing decision basis

To enhance the visualization of how the models outputted the predicted results, the CNN and Transformer models were integrated for decision basis visualization via a two-step process. The first step involved extracting the attention layers of the Transformer model, which processed the baseline and follow-up ECGs together. This extraction process resulted in a total of 24 leads, comprising 12 leads each from the baseline and follow-up ECGs. In the second step, we utilized the gradient-weighted class activation mapping method^15^ to generate an activation map. This map originated from the gradient of the final CNN layer of the Resnet50 model, which was used as our ECG embedding model. The weights derived from the first step were applied to each of the 24 leads to create the final heatmap, providing a detailed visualization of the decision-making process. Additionally, to visualize the evidence across the whole dataset rather than across individual samples, we averaged the attention map of all patients in the test dataset. Due to the variation in RR intervals among patient ECGs, we detected the peaks of the QRS complex in each ECG and then adjusted the RR intervals to 1 s, followed by an averaging process to produce an averaged attention map. A representative ECG was then overlaid on this map for display. We also utilized the local interpretable model-agnostic explanations (LIMEs) method to generate supplementary heatmaps, further enhancing the credibility of the visualizations (see Supplementary material online, *Methods*).

#### Statistics

The performance of the BNP regression model was assessed by applying Pearson’s correlation coefficient analysis. For the classification model, considering the varying sample sizes across different classes, we employed weighted metrics, including the weighted precision, weighted recall, weighted F1-score, weighted AUROC, and weighted area under the precision recall curve (AUPRC) for each class, to ensure a balanced evaluation. These weighted metrics were calculated by considering each class as a ‘one-vs-rest’ scenario, where the metric for each class was computed individually. The individual metric values were then averaged, with each value being weighted according to the number of true instances in each class. We also evaluated the overall accuracy of the model. To address potential bias due to varying test numbers per patient, we randomly selected a single test from each patient for evaluation, ensuring a fair assessment. The 95% confidence intervals (CIs) were calculated using the bootstrap method. Additionally, for determining *P* values, two-sided significance tests were utilized.

#### Software

Python 3.8 was used as the base programme, and PyTorch 1.8 was used as the deep learning package. The scikit-learn package was used to calculate the metrics.

## Results

### Patient characteristics

The study period spanned from 1 January 2012, to 31 December 2022, at the Department of Cardiology, Kobe University Hospital. A total of 6531 adult patients who underwent ECGs and BNP blood tests on the same day for each patient were eligible for inclusion. These patients had at least two such paired tests conducted on separate occasions. The average age of these patients was 64.6 years (±15.4 years), with an average BNP concentration of 66.3 pg/mL, an inter-quartile range of 24.6–175.1 pg/mL, and a left ventricular ejection fraction of 59.2% (±12.2%).

From the total cohort, patients were randomly assigned to 3 datasets: the training dataset included 4571 patients (70%), the validation dataset included 980 patients (15%), and the test dataset also included 980 patients (15%). The baseline characteristics of these groups, including age and BNP concentration ranges, are detailed in *Table 1*.

### Deep learning model for brain natriuretic peptide prediction using single 12-lead electrocardiogram

**Figure 3:**
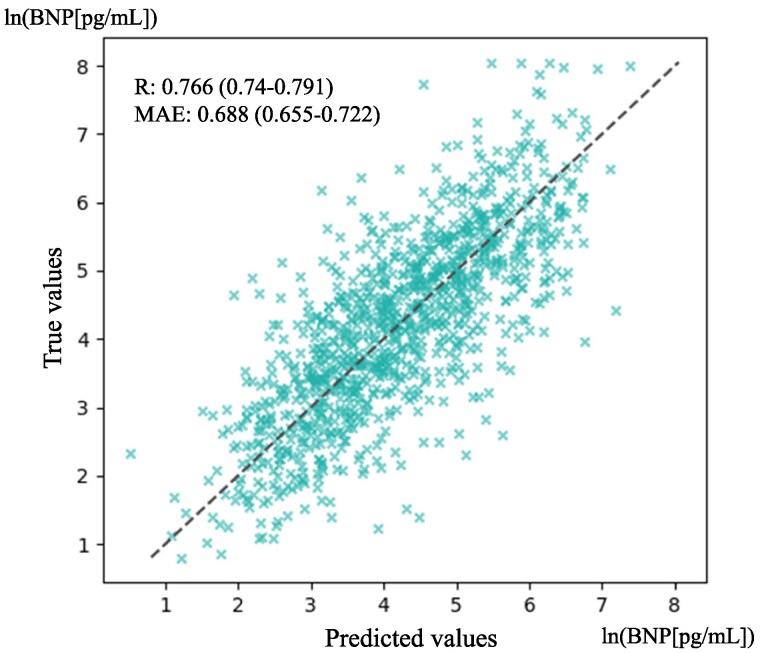
Prediction of brain natriuretic peptide with the electrocardiogram-to-brain natriuretic peptide transformer. The figure shows a scatter plot with the normalized logarithms of brain natriuretic peptide values derived from the deep learning model on the *x*-axis and the actual normalized logarithm of brain natriuretic peptide values obtained from blood tests on the *y*-axis. A substantial correlation was observed between the two variables, with a Pearson correlation coefficient of *R* = 0.766 (95% confidence interval: 0.740–0.791). The mean absolute error was calculated as 0.688 (95% confidence interval: 0.655–0.722).

We developed a deep learning model, ‘EBT’, to estimate BNP levels from single 12-lead ECG data. This model addresses the clinical need for rapid BNP estimation when immediate blood tests are unavailable. A substantial correlation was observed between the logarithms of the predicted BNP values and the actual BNP values, with a Pearson correlation coefficient of *R* = 0.766 (95% CI: 0.740–0.791). The mean absolute error was calculated as 0.688 (95% CI: 0.655–0.722). *Figure 3* illustrates the correlation between predicted and measured BNP levels on a logarithmic scale. Supplementary material online, *Figure S1* provides a focused analysis, showing the correlation between predicted and measured BNP levels on a logarithmic scale specifically for patients with left ventricular ejection fraction (LVEF) <40%.

### Dual-electrocardiogram heart failure trajectory model

Following the BNP estimation model, we developed the DEHT model to classify HF status changes using ECG data collected at two different time points. Heart failure status changes were defined as improved (≥40% decrease in BNP, baseline ≥100 pg/mL), deteriorated (≥40% increase in BNP, follow-up ≥100 pg/mL), and no-change (neither criteria were met).

**Figure 4:**
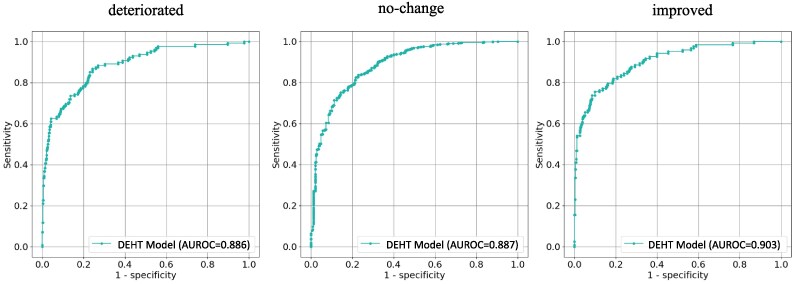
Dual-electrocardiogram heart failure trajectory model performance for heart failure status classification. Receiver operating characteristic curves of the dual-electrocardiogram heart failure trajectory model for classifying heart failure status changes. The model’s area under the receiver operating characteristic curve for each class is: deteriorated (0.886, 95% confidence interval: 0.873–0.900), no-change (0.887, 95% CI: 0.877–0.897), and improved (0.903, 95% confidence interval: 0.891–0.916). The model uses paired electrocardiogram data to classify heart failure progression.

The DEHT model classifies HF status changes directly from ECG data without individual BNP estimations. In the test dataset, the DEHT model achieved an AUROC of 0.889 (95% CI: 0.879–0.898) and an accuracy of 0.871 (95% CI: 0.864–0.878). These results indicate the ability of the model to identify changes in HF status using ECG data collected at two different time points. The class-specific AUROCs are shown in *Figure 4*. The detailed performance metrics are in *Table 2*. Additional subgroup analyses were conducted to evaluate the DEHT model’s performance in specific patient populations. For patients with BNP ≥100 pg/mL, the results are summarized in Supplementary material online, *Table S1*. Similarly, for patients with LVEF <40%, the detailed metrics are provided in Supplementary material online, *Table S2*.

**Table 2:** Performance metrics of dual-electrocardiogram heart failure trajectory model developed by AI and 12-lead electrocardiogram waveform signals in the test dataset

	Accuracy	Precision	Recall	Specificity	AUROC	AUPRC	F1-score
Deteriorated	0.916 (0.909–0.923)	0.633 (0.486–0.846)	0.784 (0.696–0.857)	0.900 (0.826–0.972)	0.886 (0.873–0.900)	0.653 (0.618–0.685)	0.695 (0.614–0.779)
Improved	0.926 (0.919–0.932)	0.201 (0.144–0.318)	0.947 (0.800–1.000)	0.712 (0.624–0.883)	0.903 (0.891–0.916)	0.708 (0.674–0.740)	0.327 (0.252–0.457)
No-change	0.855 (0.846–0.863)	0.952 (0.936–0.965)	0.836 (0.802–0.883)	0.865 (0.812–0.905)	0.887 (0.877–0.897)	0.955 (0.950–0.961)	0.890 (0.872–0.913)
Overall	0.871 (0.864–0.878)	0.871 (0.855–0.888)	0.868 (0.855–0.886)	0.762 (0.728–0.798)	0.889 (0.879–0.898)	0.888 (0.880–0.898)	0.868 (0.853–0.885)

Performance of DEHT model across different classes—‘deteriorated’, ‘improved’, ‘no-change’—and an overall evaluation incorporating all these classes. Values in the table are reported with their respective 95% CIs. DEHT model: combines baseline and follow-up ECGs into a 24-lead set for single inference, directly classifying HF status.

AUROC, area under the receiver operating characteristic curve; AUPRC, area under the precision recall curve.

### Attention mapping

**Figure 5:**
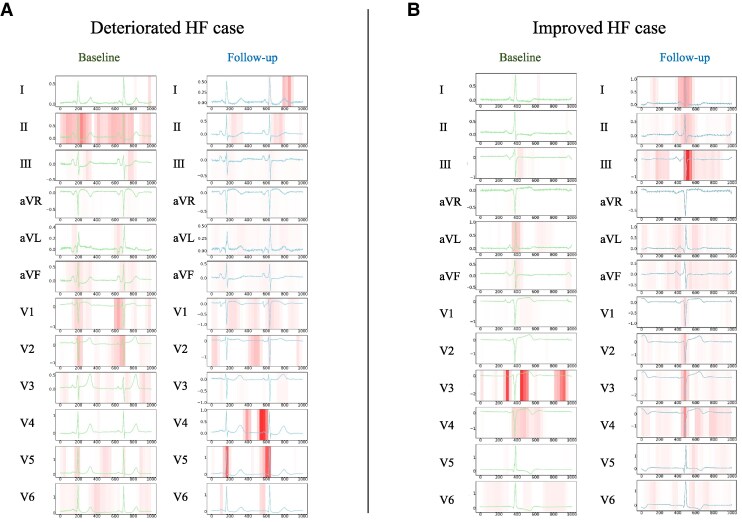
Diagnostic basis visualization: representative cases. (*5A*) Twelve-lead baseline and follow-up electrocardiograms of the same patient, with highlighted areas denoting segments that contribute to the detection of a deteriorated class. The model was strongly activated by the QRS complex in Lead V5, as well as by the P wave in Lead V4. (*5B*) Twelve-lead baseline and follow-up electrocardiograms of the same patient with an applied attention map. The highlighted areas represent the segments that activated the model towards the prediction of an improved class. The model was particularly activated by increased R-wave amplitude in the pre-cordial leads.

**Figure 6:**
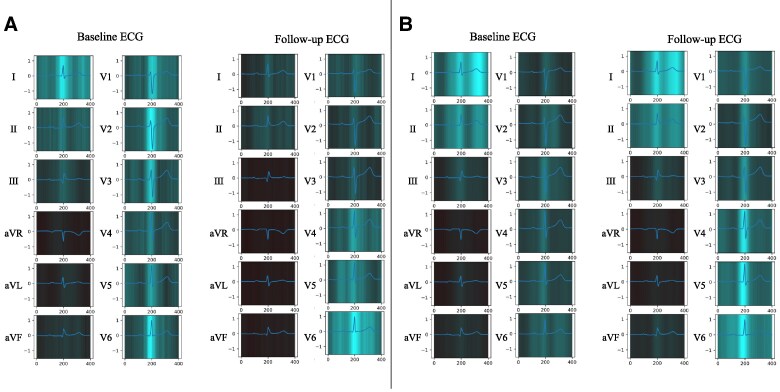
Diagnostic basis visualization: averaged attention mapping. (*A*) The averaged attention map for the deteriorated class. (*B*) The averaged attention map for the improved class. We visualized the averaged attention maps for all samples within each class of the test dataset. Given the variability in heart rate among the electrocardiograms, we standardized the RR intervals to be 1 s consistently and adjusted the lengths before averaging the attention map values for each of the 12 leads. The electrocardiograms overlaid on the attention maps are not the average electrocardiograms of all samples. Instead, they are representative electrocardiograms selected specifically to clearly illustrate the positions of the P waves, QRS complexes, and T waves.

To visualize our models’ diagnostic features, we combined attention maps with the GradCAM method. This analysis focused on two important aspects: representative cases from deteriorated and improved HF groups and an averaged attention map across all patients in the test dataset for each class. *Figure 5A* and *5B* show baseline and follow-up 12-lead ECGs of patients with either deteriorated or improved HF status. The relative significance of the extracted features varied across the patients tested. According to the averaged attention map across the entire patient ECGs for each class, the QRS complex was the most important feature for determining model outputs with smaller effects seen for P waves and T waves variance (*Figure 6*). The results derived from the LIME method are provided in the Supplementary material online, *Figure S2*-*1* and *S2-2*.

## Discussion

We developed and evaluated Transformer-based deep learning models, specialized for 12-lead ECG analysis. The primary outcomes of our study included the following: (i) the ability of the EBT model to accurately estimate BNP levels from ECG data, as indicated by a correlation coefficient of 0.766 (95% CI: 0.740 –0.791), (ii) the development of the DEHT model for HF status identification, demonstrating an AUROC of 0.889 (95% CI: 0.879–0.898) and an accuracy of 0.871 (95% CI: 0.864–0.878); and (iii) the attention mapping analysis that emphasized the significance of the QRS complex in the models’ decision-making, as shown in *Figures 5* and *6*. These findings collectively highlighted the effectiveness of deep learning in cardiological assessments and personalized approaches for managing HF.

To our knowledge, this is the first model that examines the temporal changes in ECGs, demonstrating its effectiveness and high accuracy in classification, comparable with BNP for HF assessment. Previous deep learning models predict asymptomatic left ventricular ejection fraction decline (AUROC = 0.93), left ventricular dysfunction (AUROC = 0.83), and right ventricular dysfunction (AUROC = 0.84). However, the ability of these models to maintain predictive accuracy over multiple assessments through time was uncertain, as they forecast outcomes from a single point in time. The DEHT model overcame this challenge by analysing ECGs from two distinct time points concurrently, thus avoiding the complexity of multiple analyses and eliminating individual variations such as body mass index, achieving an AUROC of 0.889 (95% CI: 0.879–0.898) in identifying exacerbation or alleviation of HF. This performance is comparable with that of previous deep learning models. To optimize performance for HF monitoring, our algorithm used readily available ECG waveform data collected from a large number of patient cohorts.

The DEHT model effectively minimized the impact of patient-specific factors such as body mass index, age, and anatomical axis variations, using the historical ECG data from the same individual. This approach improved the ability of the model to identify relevant ECG changes associated with HF progression. Owing to the importance of visualizing diagnostic rationales in deep learning models, we employed attention mapping for visualization. Two types of attention analyses were conducted: first, we used averaged attention maps for each class in the test dataset; second, we conducted attention mapping on specific cases, focusing on representative examples from both the deteriorated and improved HF groups. Averaged attention mapping highlighted the significant role of the QRS complex in detecting HF changes, with less emphasis on P or T waves (*Figure 6*). A detailed analysis of individual cases demonstrated that the ability to identify important diagnostic regions in the ECG varied across different cases. In *Figure 5A*, the model emphasizes the P wave and the QRS complex in the follow-up ECG. *Figure 5B* shows that attention is focused on the QRS complex amplitude. These findings are consistent with prior studies linking ECG waveforms to HF indicators.^16–18^ While these observations provide valuable insights, the interpretability of attention mapping outputs remains limited, as noted in the ‘Limitations’ section.

The DEHT model is characterized by its non-invasive nature and cost-effectiveness, which enhances early detection and continuous monitoring of HF, potentially improving outcomes through early intervention.^5,7^ In scenarios where only a single ECG is available, the EBT model provides a complementary alternative for estimating BNP levels.

In areas where BNP testing is unavailable due to outsourced laboratory services or regional limitations, the DEHT model, which operates independently of baseline or follow-up BNP measurements, serves as an alternative solution to detect important ECG changes for earlier treatment. This approach highlights the complementary roles of BNP and ECG in clinical practice, with ECG serving as a valuable modality for monitoring HF dynamics when biochemical data are not directly available. By including participants without a confirmed HF diagnosis, the DEHT model is designed to detect both HF status changes and new-onset HF, while also accounting for cases where HF is suspected but ultimately ruled out. This approach ensures the model reflects real-world clinical scenarios, where HF diagnosis is not always established at baseline. As a result, the model achieves broader applicability and enhances its potential utility in diverse healthcare settings. While not a replacement for echocardiograms, the model could decrease the dependence on continuous HF assessment, thus reducing patient burden and healthcare costs.

Future research should focus on developing multimodal models incorporating conventional patient characteristics and risk factors as input as well as ECG data. This approach could overcome current limitations and provide a more comprehensive assessment of HF progression. Expanding the study population to include individuals undergoing health check-ups could broaden the model’s applicability. Recent studies have demonstrated promise for both BNP and ECG in population screening settings.^19,20^ With appropriate modifications, our model could potentially be adapted for use in broader patient populations, including those in health screening scenarios.

### Limitations

This study has several limitations. First, BNP levels can be influenced by factors such as renal function and obesity.^21^ Rapid changes in these factors might reduce the accuracy of the model. Our study excluded patients with conditions known to significantly affect BNP levels independent of HF status, which may limit the generalizability of our findings to these populations.

Second, the research was conducted in a single facility, potentially introducing sample bias. Additionally, the dataset’s limited variability in patient characteristics may increase the risk of overfitting when using a ResNet50-based model. Future studies should include a wider range of participants and multicentre settings to validate the model’s performance across diverse patient populations and clinical scenarios.

Third, AI-based models, including the DEHT model, are inherently limited by their black-box nature, which poses challenges in transparency and explainability. While methods such as attention mapping can provide insights, they may not fully resolve these limitations.^22^

## Conclusions

Deep learning analysis of two-point ECG data effectively identified changes in HF status. The performance of our model suggests its potential as a foundational tool for advancing HF monitoring and management strategies.

## Supplementary Material

ztaf016_Supplementary_Data

## Data Availability

The data that support the findings of this study are not publicly available due to ethical and legal restrictions. However, they are available from the corresponding author upon reasonable request and with permission from the Ethics Committee of Kobe University Hospital.
