# Supplementary material for: Identifying heart failure dynamics using multi-point electrocardiograms and deep learning
Source: Eur Heart J Digit Health. 2025 Mar 10;6(3):447–55. doi: 10.1093/ehjdh/ztaf016 (PMC12088712; doi:10.1093/ehjdh/ztaf016)
Supplement: ztaf016_Supplementary_Data [file ztaf016_supplementary_data.docx]

Supplemental methods: Visualizing Decision Basis with LIME method

To enhance the reliability of the model’s interpretability, we validated the predictions using the Local Interpretable Model-agnostic Explanation (LIME) method, ^1^ in addition to other visualization techniques.

LIME explains the predictions of any classifier by fitting a linear regression model locally around the prediction. For each sample, the following steps were performed:

1. The 2-second ECG data was divided into 20 equal segments (1 segment = 50 points). Each segment was randomly masked with zero values with a 50% probability and input into the trained model.

2. A distance $d\left( x, x^{'} \right)$ between the original and masked samples was calculated based on the proportion of the image mask applied. The weighting function$w\left( x, x^{'} \right)$was then computed using the formula:

$$w\left( x,x^{'} \right)=exp \left( -\frac{d\left( x, x^{'} \right)^{2}}{\sigma^{2}} \right)$$

where $\sigma$ was set to 0.25. This weighting function ensures that the linear model focuses on samples that are more representative of the original data while reducing the influence of outliers or highly perturbed samples.

3. This process was repeated 1,000 times, and for each segment, the following equation was solved to compute $\beta$:

$$\min\beta\sum_{i=1}^{N} w\left( x, x_{i}^{'} \right) \cdot\left( f\left( x_{i}^{'} \right) - g\left( x_{i}^{'}, \beta\right) \right)^{2}$$

Here, $g\left( x^{'},\beta\right)$ represents a linear regression model that approximates the output of the trained model $f\left( x^{'} \right)$ within the local neighborhood of the original sample x. By solving this equation, the linear model $\beta$ is fit to the predictions of the trained model in the perturbed data space. The absolute value of $\beta$ was interpreted as the contribution of each segment to the prediction.

4. Finally, the contributions of all segments for each sample were standardized and visualized as a heatmap, providing an intuitive representation of the model’s decision basis for each sample.

Supplemental Table 1: performance metrics of dual-ECG heart failure trajectory model (DEHT model) developed by AI and 12-lead ECG waveform signals in test dataset for patients with BNP ≥ 100 pg/ml

|  | **Accuracy** | **Precision** | **Recall** | **Specificity** | **AUROC** | **AUPRC** | **F1-Score** |
| --- | --- | --- | --- | --- | --- | --- | --- |
| **deteriorated** | 0.836 (0.804–0.876) | 0.738 (0.640–0.923) | 0.722 (0.614–0.800) | 0.884 (0.829–0.981) | 0.803 (0.778–0.830) | 0.772 (0.725–0.836) | 0.724 (0.682–0.767) |
| **improved** | 0.861 (0.771–0.928) | 0.597 (0.411–0.793) | 0.820 (0.714–0.929) | 0.870 (0.746–0.961) | 0.844 (0.817–0.877) | 0.724 (0.658–0.802) | 0.678 (0.564–0.783) |
| **no-change** | 0.792 (0.771–0.817) | 0.785 (0.747–0.822) | 0.839 (0.802–0.902) | 0.740 (0.667–0.789) | 0.789 (0.767–0.813) | 0.855 (0.836–0.875) | 0.811 (0.785–0.836) |
| **overall** | 0.822 (0.803–0.840) | 0.785 (0.761–0.810) | 0.755 (0.732–0.778) | 0.803 (0.778–0.826) | 0.867 (0.847–0.886) | 0.830 (0.804–0.853) | 0.760 (0.735–0.786) |

Performance of DEHT model across different classes – 'deteriorated', 'improved', 'no-change'– and an overall evaluation incorporating all these classes in patients with BNP ≥ 100 pg/ml. Values in the table are reported with their respective 95% CIs. Abbreviations used: AUROC, Area Under the Receiver Operating Characteristic Curve; AUPRC, Area Under the Precision Recall Curve.

DEHT model: Combines baseline and follow-up ECGs into a 24-lead set for single inference, directly classifying heart failure status.

Supplemental Table 2: performance metrics of dual-ECG heart failure trajectory model (DEHT model) developed by AI and 12-lead ECG waveform signals in test dataset for patients with LVEF < 40%

|  | **Accuracy** | **Precision** | **Recall** | **Specificity** | **AUROC** | **AUPRC** | **F1-Score** |
| --- | --- | --- | --- | --- | --- | --- | --- |
| **deteriorated** | 0.820 (0.782–0.855) | 0.768 (0.667–0.867) | 0.699 (0.600–0.800) | 0.886 (0.824–0.946) | 0.791 (0.748–0.837) | 0.786 (0.730–0.839) | 0.729 (0.667–0.789) |
| **improved** | 0.858 (0.782–0.927) | 0.516 (0.364–0.714) | 0.802 (0.714–1.000) | 0.868 (0.761–0.957) | 0.834 (0.784–0.890) | 0.676 (0.584–0.765) | 0.624 (0.500–0.737) |
| **no-change** | 0.798 (0.764–0.836) | 0.837 (0.783–0.905) | 0.747 (0.680–0.833) | 0.851 (0.800–0.920) | 0.798 (0.763–0.837) | 0.857 (0.820–0.893) | 0.790 (0.745–0.836) |
| **overall** | 0.779 (0.747–0.813) | 0.745 (0.706–0.790) | 0.709 (0.673–0.745) | 0.768 (0.720–0.808) | 0.852 (0.818–0.884) | 0.807 (0.767–0.848) | 0.707 (0.667–0.751) |

Performance of DEHT model across different classes – 'deteriorated', 'improved', 'no-change'– and an overall evaluation incorporating all these classes in patients with LVEF <40%. Values in the table are reported with their respective 95% CIs. Abbreviations used: LVEF, left ventricular ejection fraction; AUROC, Area Under the Receiver Operating Characteristic Curve; AUPRC, Area Under the Precision Recall Curve.

DEHT model: Combines baseline and follow-up ECGs into a 24-lead set for single inference, directly classifying heart failure status.

Supplemental Figure 1: Prediction of BNP with the ECG-to-BNP Transformer (EBT) for Patients with LVEF < 40%


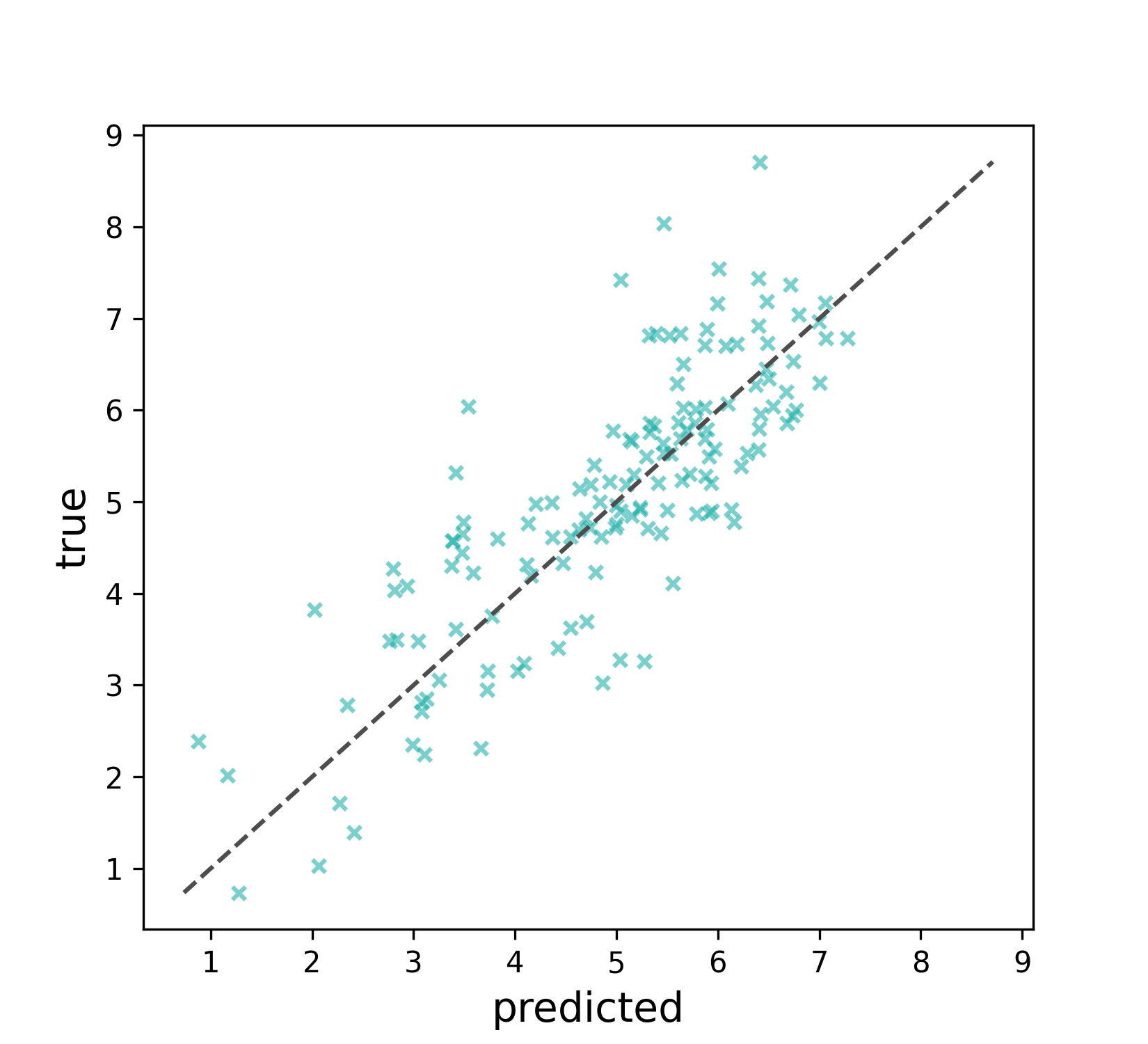


True values

ln(BNP[pg/mL])

ln(BNP[pg/mL])

R: 0.814 (0.749-0.864)

MAE: 0.679 (0.592-0.767)

Predicted values

The figure shows a scatter plot with the normalized logarithms of brain natriuretic peptide (BNP) values predicted by the deep learning model (x-axis) and the actual normalized logarithm of BNP values obtained from blood tests (y-axis) for patients with LVEF <40%. A substantial correlation was observed between the two variables, with a Pearson correlation coefficient of R = 0.814 (95% CI: 0.749–0.864). The mean absolute error (MAE) was calculated as 0.679 (95% CI: 0.592–0.767).

Supplemental Figure 2: Diagnostic Basis Visualization Using the LIME Method – Representative Cases

2-1: Deteriorated HF Case (Corresponding to Figure 5A)


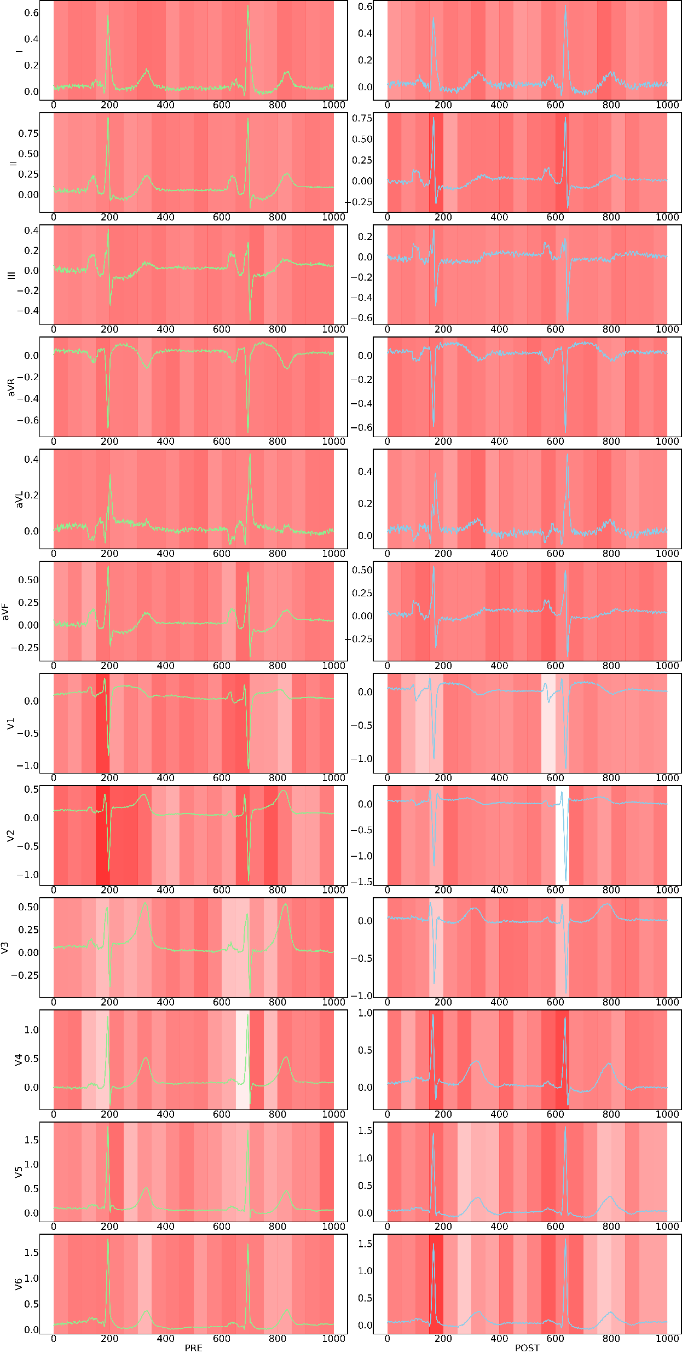


Twelve-lead baseline (left) and follow-up ECGs (right) from the same patient. Red-highlighted areas indicate segments contributing to the classification of the deteriorated class. The model exhibited strong activation in the QRS complex, particularly in Leads V1 and V2 in the baseline ECGs, and in Leads II, V4, and V6 in the follow-up ECGs.

2-2: Improved HF Case (Corresponding to Figure 5B)


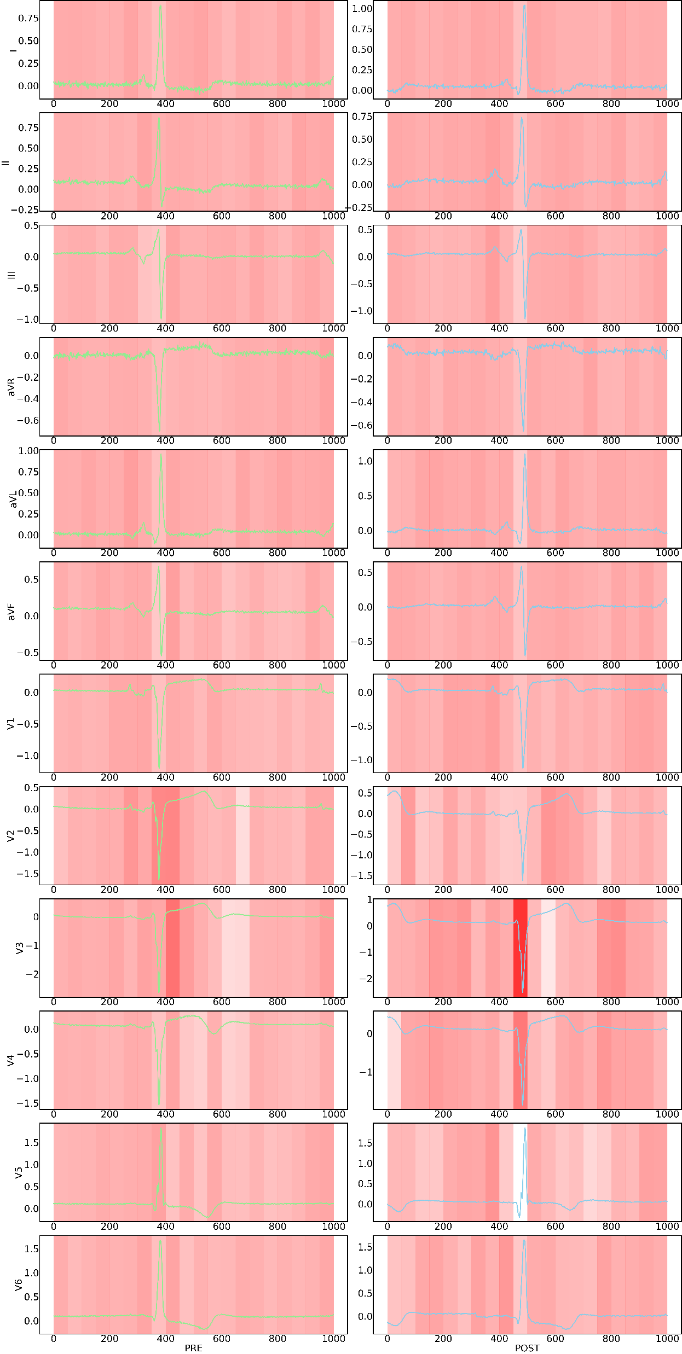


Twelve-lead baseline (left) and follow-up ECGs (right) from the same patient. Red-highlighted areas indicate segments contributing to the classification of the improved class. The model showed notable activation in the QRS complex, particularly in Leads V3 and V4 in the follow-up ECGs.

References:

1. Ribeiro M, Singh S, Guestrin C. "Why Should I Trust You?": Explaining the Predictions of Any Classifier; 2016.
